# Gaps and Pathways to Success in Global Health Informatics Academic Collaborations: Reflecting on Current Practices

**DOI:** 10.2196/67326

**Published:** 2025-11-28

**Authors:** Elizabeth A Campbell, Felix Holl, Oliver J Bear Don't Walk IV, Badisa Mosesane, Andrew S Kanter, Hamish Fraser, Amanda L Joseph, Judy Wawira Gichoya, Kabelo Leonard Mauco, Sansanee Craig

**Affiliations:** 1 Johns Hopkins Center for Outbreak Response Innovation, Baltimore, MD, United States; 2 DigiHealth Institute, Neu-Ulm University of Applied Sciences, Wileystraße 1, Neu-Ulm, BY, 89231, Germany, 49 7319762 ext 1613; 3 Leibniz ScienceCampus Digital Public Health, Bremen, Germany; 4 Department of Biomedical Informatics and Medical Education, University of Washington, Seattle, WA, United States; 5 Department of Computer Science, University of Botswana, Gaborone, Botswana; 6 Department of Biomedical Informatics, Columbia University, New York, NY, United States; 7 Brown Center for Biomedical Informatics, Brown University, Providence, RI, United States; 8 School of Health Information Science, University of Victoria, Victoria, BC, Canada; 9 Department of Learning, Informatics, Management and Ethics, Karolinska Institutet, Solna, Sweden; 10 Department of Biomedical and Health Informatics, Children's Hospital of Philadelphia, Philadelphia, PA, United States; 11 Department of Biomedical Informatics, Columbia University Irving Medical Center, New York, NY, United States

**Keywords:** global health informatics, academic collaboration, global health, digital health, health equity, academic, high-income countries, low- and middle-income countries, LMIC, GHI, research community, participatory research, development, sustainability, scalability, GHI intervention, eHealth

## Abstract

Academic global health informatics (GHI) projects are impactful collaborations between institutions in high-income and low- and middle-income countries (LMICs) and play a crucial role in enhancing health care services and access in LMICs using eHealth practices. Researchers across all involved organizations bring unique expertise to these collaborations. However, these projects often face significant obstacles, including cultural and linguistic barriers, resource limitations, and sustainability issues. The lack of representation from LMIC researchers in knowledge generation and the high costs of open-access publications further complicate efforts to ensure inclusive, accessible, and collaborative scholarship. This viewpoint describes present gaps in the literature on academic GHI collaborations and describes a path forward for future research directions and successful research community development. Key recommendations include centering community-based participatory research, developing post-growth solutions, and creating sustainable funding models. Addressing these challenges is essential for fostering effective, scalable, and equitable GHI interventions that improve global health outcomes.

## Introduction

Global health informatics (GHI) is an interdisciplinary subfield of health informatics that aims to use eHealth practices (eg, electronic health records, mobile health [mHealth], telehealth, and technology-enabled learning), including research and innovation in enhancing provision of health care resources, services, including information access in low- and middle-income countries (LMICs). This approach has the potential to improve clinical and population health outcomes for patients in LMICs [1,2]. GHI interventions aim to empower people through context-specific technological solutions to develop innovative solutions that improve health care access and patient outcomes while being locally led and maintained [3].

Academic GHI interventions are research-driven initiatives between higher education institutions (eg, universities and medical schools) with host country governmental agencies, nongovernmental organizations, universities, and community organizations [4,5]. These projects typically have more localized and regional scope, focusing on specific local issues within a particular community, whether in high-income or low-income settings, systems, or regions; they often address local needs without prioritizing global scalability or broad collaboration. Academic GHI collaborations can include an informatics education training component to build local capacity [6,7]. Finally, a key characteristic of academic GHI projects is that their funding typically comes from governmental agencies or foundations for institutional resources. Knowledge generation through scientific publications and obtaining maintained extramural funding are key differentiating factors between academic collaborations and other GHI interventions.

However, numerous challenges impact the long-term success of academic GHI interventions and collaborations. These include a lack of understanding of the true health care problems, goals, and workflows, “pilotitis” (ie, the lack of scale-up beyond a pilot phase), and insufficient resources or interest amongst communities to maintain interventions independently [8-10]. There is a substantial lack of representation from LMIC researchers in knowledge generation and a scarcity of literature on successful implementation and further operationalization of academic GHI interventions [11,12]. Despite the benefits of open-access publication for knowledge sharing in LMICs, there is a substantial variance in open-access article processing charges, with costs upwards of several thousand US dollars; this limits access to and dissemination of scientific knowledge from researchers in LMICs and globally [13].

While the phenomenon of “pilotitis” remains a recognized challenge in GHI, it does not universally reflect the realities on the ground. In fact, there are numerous examples of successful, long-term implementations of digital health technologies in Africa and Southeast Asia that have benefited from international collaboration while being adapted to local needs.

A leading example is OpenMRS, an open-source electronic medical record system that has been deployed and maintained across multiple countries, including Kenya, Rwanda, Uganda, and Bangladesh [14,15]. These implementations have demonstrated local ownership, strong technical communities, and policy alignment, proving that long-term sustainability is achievable when supported by appropriate governance and capacity building.

Another widely embraced platform is District Health Information Software 2, developed by the University of Oslo and implemented in over 75 countries [16]. It serves as the national health information system (HIS) in countries such as Tanzania, Nepal, and Sri Lanka, enabling robust health data management and public health decision-making at scale. District Health Information Software 2’s success is rooted in its open architecture, local customization, and integration into national health strategies.

These examples highlight that international collaboration can indeed support scalable, sustainable innovation, particularly when it promotes local leadership, long-term investment, and codevelopment. Rather than suggesting that pilotitis is a universal outcome, we aim to emphasize the importance of structural enablers and collaboration models that move beyond fragmented, short-term interventions and foster enduring digital health ecosystems.

In this viewpoint, we describe current gaps in the literature on how academic GHI collaborations can be successfully implemented. Our viewpoint also explores a path forward for developing an environment that is conducive to these projects’ success, and research priorities for the informatics community to expand academic GHI research. The authors would like to call attention to the use of terms such as “LMICs” throughout this piece. To foster effective global collaboration, respect for the country of origin’s culture, customs, and economy is warranted. Such a perspective could place unwarranted stigma or an unequal distribution of power or influence on countries in the southern hemisphere, implying that they are unable to innovate and must remain reliant on high-income countries for solutions to local, country-specific challenges [17,18]. In the absence of alternatives, we continue to use the current nomenclature but recognize it as a limitation and a priority area for developing more inclusive terms.

## Expanding the Knowledge Base for Academic GHI Project Implementation

### Overview

Although academic GHI interventions are typically implemented locally, they often adopt a more expansive viewpoint by striving to contribute scientific knowledge for solutions to be applied globally. Long-term collaborations across academic institutions and other health care organizations globally are key to technological advancements and ensuring that innovations are scalable and transferable across diverse health contexts [11]. For example, interoperable systems and standards, such as Health Level Seven International, Fast Healthcare Interoperability Resources and Systematized Nomenclature of Medicine–Clinical Terms, are essential to ensuring that health information can easily be exchanged, interpreted, and used across various systems worldwide [19]. Interoperability systems and standards are essential for ensuring secure health information exchange, facilitating data analysis and secondary research use, informing organizational decision-making, and improving patient care.

Despite the critical role of GHI in advancing health care outcomes worldwide, there remains a significant gap in the literature regarding the specific challenges faced during the implementation and scale-up of such projects in LMICs. This has often led to “pilotitis,” where systems are designed and implemented yet never scale or report evaluation findings beyond the initial implementation [8,12]. These challenges can be grouped into 3 categories: local context-related, resource-related, and business model-related.

### Local Context–Related Challenges

Local context–related challenges encompass cultural and linguistic barriers that can lead to the development of solutions that are not suitable or compatible with the target population. For example, health data may be recorded in different languages, resulting in complicated and potentially erroneous translations, thus challenging data standardization and interoperability. Similarly, existing medical diagnostic codes, terms, and classifications may not align with existing languages and across countries and cultures. These barriers can result in a lack of user engagement and adoption, ultimately limiting the intervention’s effectiveness. These challenges can also include data privacy and security concerns, as well as divergent cultural views and perceptions of diseases and health behaviors, and trust in technology. Understanding and integrating local cultural norms, language requirements, and workflows are crucial for health informatics initiative success [20].

### Resource-Related Challenges

Resource-related challenges include technological infrastructure limitations, but extend beyond those and include the availability of a skilled workforce. There is often a shortage of trained professionals (ie, technical and clinical) who can manage and maintain digital health systems, analyze health data, and ensure data security and privacy. This lack of expertise can lead to improper or inappropriate technologies, security vulnerabilities, a lack of data access and use, and unreliable data, which undermine interventions’ goals [20]. To increase capacity, additional training of local staff is often necessary, a hidden cost in time and money that may not always be accounted for in grant funding or limited project timelines for academic GHI interventions. Furthermore, even in initiatives that include data science education in their activities, there is a lack of standardization in capacity-building approaches, which is needed to support global and interdisciplinary collaborations and foster strong regional partnerships [21].

### Business Model–Related Challenges

Business model–related challenges involve the sustainability and scalability of health informatics projects. Projects often rely on short-term grants or donor funding and lack a long-term financial sustainability plan; this issue is further compounded by challenges faced in communicating return on investment for GHI projects to funders (eg, governments and private donors), particularly given limitations in integrating cost-recovery mechanisms into these projects due to infeasibility or ethical considerations. In addition, vague ownership models for data, systems, and technologies can limit scale-up and integration of smaller-scale informatics project products into national health systems. This issue highlights the need for robust business models that ensure continuous support, funding, and integration of digital health interventions into the broader health system framework [22,23].

In addition, there is limited analysis of how projects navigate these hurdles in real-world settings beyond the pilot stage or the effectiveness of various strategies to overcome these obstacles. While in recent years, there have been some studies on evaluating GHI projects in LMICs [14,24,25], more comprehensive evaluations and reporting on the implementation processes and intervention outcomes at scale are necessary to address these gaps. Such analyses can provide valuable insights into best practices and successful strategies for overcoming the myriad challenges faced in low-resource settings. Studies need to examine these projects’ immediate impacts, along with their ability to be integrated into existing health systems, address local community needs, adaptability to changing technological landscapes and health challenges, and capacity to remain relevant and useful over time [26,27]. Centrally positioning LMIC researchers as experts in knowledge generation is imperative, as is acknowledging and mitigating harmful power dynamics between researchers from academic institutions and members of communities in which GHI projects are situated [28,29]. Addressing these gaps is essential for guiding future academic GHI collaborations toward more successful, sustainable, and impactful implementation, thereby contributing to improving global health outcomes.

## Developing an Environment That Fosters Success

Global efforts to establish an enabling environment for academic GHI interventions are necessary. In such an environment, stakeholders are invested in GHI projects as a solution to address health care challenges and are prepared to engage in ethical and mutually beneficial informatics projects. As mutually beneficial collaborations, researchers in high-resource settings can benefit greatly from the expertise and interventions proven successful in low-resourced settings. For example, in response to an urgent need for COVID-19 tracking early in the pandemic, the San Francisco Department of Health partnered with Dimagi to use CommCare, an open-sourced tracking application used for 2 decades by community health workers in low-resource settings [30,31]. This partnership led to the successful expansion of the city’s COVID-19 response force and scale-up of testing capabilities, forming a generalizable model for other United States jurisdictions [30].

Furthermore, successful academic GHI interventions generally require more input and control from local communities compared to typical commercial HIS seen in the US and other high-income settings. Developing interventions with community buy-in and local ownership is critical to addressing these challenges and should be incorporated into protocols and implementation efforts from a study’s inception [24]. For instance, systems must function with limited power, networking, and IT support [32]. Key solutions for developing and implementing successful GHI projects in low-resource settings include flexible coding systems (eg, the Open Concept Lab for OpenMRS [33]) to represent locally important data on diseases, treatments, and social circumstances, such as transport and housing types, along with a focus on open and rigorous evaluation in clinical contexts where informatics solutions are mobilized.

Evaluating and establishing a new program needs to be driven by the equitable involvement of all relevant parties with a mutual and unified understanding of goals and pathways to be achieved. Theories and frameworks regarding technology adoption, sociocultural, user-centered design, and implementation science are important tools to help guide this process [4,34].

While our perspective highlights persistent challenges in GHI academic collaborations, we recognize and seek to build upon the substantial efforts of leading organizations and institutions. International academic partnerships around GHI have played an important role in strengthening health systems, especially in LMICs.

The World Health Organization has played a critical role in shaping the global digital health agenda through its Global Strategy on Digital Health, the Digital Health Technical Advisory Group, and its coordination of normative frameworks and implementation support mechanisms. The Geneva University Hospitals and the Geneva Digital Health Hub have emerged as key drivers of innovation, knowledge exchange, and capacity building. As a World Health Organization Collaborating Center for eHealth and Telemedicine, Geneva University Hospitals has contributed extensively to advancing digital health solutions for global health challenges. The Geneva Digital Health Hub has further expanded this impact by convening stakeholders across sectors, translating research into policy, and facilitating collaborative digital health projects with a strong emphasis on equity and inclusion.

Other notable examples include the Academic Model Providing Access to Healthcare and the International Training and Education Center for Health at the University of Washington. These collaborations underscore the potential of cross-institutional efforts in driving sustainable health improvements through shared expertise, coleadership, and a better understanding of local contexts. One such partnership that has emerged as a promising model is the collaboration between the Children’s Hospital of Philadelphia (CHOP) and the University of Botswana (UB) on GHI research efforts, which is described as a case study in detail.

## Case Study: Chop-UB Partnership—a Model for Equitable Global Health Collaboration

The CHOP-UB partnership exemplifies a coleadership model built on mutual respect, shared responsibilities, and the integration of diverse expertise. CHOP brings extensive experience in pediatric health care delivery and clinical research, while UB contributes technical and contextual knowledge critical to health care delivery in Botswana. Specifically, researchers and experts at UB engage in data management and analysis through the eHealth research unit, focusing on ensuring data quality and usability. Their expertise spans digital health technologies, including electronic health records and mHealth applications, as well as compliance with ethical and regulatory frameworks governing data privacy in Botswana.

Key achievements of this collaboration include: the evaluation of health care data collection, sharing, and usage, mapping the flow of health care data, examining clinicians’ perspectives on health care data management, and data science capacity development. The CHOP-UB collaboration is focused on building capacity through comprehensive training programs, which are designed and customized for local Botswana health care professionals’ needs. These programs emphasized the use of health informatics tools, and the importance of data-driven decision-making in clinical settings. Key factors in this academic GHI partnership’s success have been a focus on local context (eg, socioeconomic, cultural, and geographic), use of robust evaluation frameworks [35], focus on long-term outcomes, scalability, and consideration of mitigating barriers to informatics tools adoption, infrastructure deficits, and resistance to change.

A defining characteristic of the partnership has been the commitment to knowledge transfer. There has been a particular focus on knowledge transfer to empower local staff to independently manage and maintain technological advancements, thereby ensuring long-term impact and sustainability. Rather than creating dependency, the programs are designed to empower local health care workers to independently manage and adapt digital tools to meet the evolving needs of the local health system. This approach is critical for ensuring sustainability and meaningful impact. The partnership has adopted a coleadership model where leaders from both CHOP and UB bring skills and expertise ensuring local health care staff are actively involved in capacity building to achieve this knowledge transfer.

Botswana is currently making strides in implementing advanced health informatics solutions to enhance the quality of health care services across facilities. Notable efforts include electronic medical record rollouts, revitalizing primary health care systems, and the development of the health information exchange and the Botswana eHealth Enterprise Architecture. These efforts are geared toward improving patient outcomes, streamlining data management, and facilitating near-real-time clinical decision-making. The CHOP-UB academic partnership’s success exemplifies the potential benefit of international collaborations in transforming health care delivery through innovative informatics solutions in low-resource settings [36].

Other stellar examples of collaborative international health informatics efforts include those led by Japan to address infectious diseases prevalent in developing nations through the Global Health Innovative Technology Fund, a public-private partnership [37], and through SATREPS (Science and Technology Research Partnership for Sustainable Development) [38]. In addition, regional partnerships among LMICs (sometimes termed “South-South”) in health informatics include those among South and Southeast Asian countries, sub-Saharan African nations [39,40], and Latin American nations [7]. These efforts demonstrate the value of cross-national strengthening of HIS to promote global health outcomes. The 2022 International Medical Informatics Association Yearbook contains the most recent updates on the status of health informatics societies around the world, including Asian, Latin American, and African nations [41,42].

## Lessons Learned

This case study offers several important lessons for academic GHI partnerships.

1. Contextual relevance is vital: successful collaborations require a deep understanding of local health system needs, real-life workflows during patient encounters, cultural dynamics, and HIS infrastructure realities. Tailoring solutions to these conditions has the potential to enhance adoption and sustainability.
2. Coleadership builds trust and equity: shared leadership between institutions promotes equitable partnerships and ensures both global and local stakeholders contribute meaningfully to projects and decision-making.
3. Capacity building enables sustainability: investing in local capacity through knowledge transfer and educational initiatives supports sustainable implementation. Intentionally designing knowledge transfer promotes maintenance skills and future innovation of health information technology by local experts and clinical champions.
4. Evaluation strengthens accountability: using robust evaluation frameworks not only measures impact but also guides program adaptation and scalability.
5. Infrastructure and policy alignment are key: tech solutions are more effective when aligned with national strategies and supported by strong policy environments.

## Future Research Directions

GHI academic collaborations can undoubtedly draw on HIS technology intervention strengths in high-income settings; however, opportunities to truly meet the needs of diverse global health systems remain. Potential future directions include (1) centering community-based participatory research (CBPR) approaches to colead research with local experts, (2) embracing a post-growth mentality focused on quality of life and equitable distribution of technological benefits (using technology to improve quality and reach of existing health systems rather than solely focusing on growth) to develop GHI interventions, and (3) supporting frameworks for evaluation and scaling that address how to move beyond “pilotitis,” and toward sustainable technology implementation and use.

CBPR approaches promote effective, long-term, and equitable collaborations between research teams and community-based organizations with community members representing underserved groups [43]. Fundamentally, the approach centers end-users from marginalized communities in all stages of research, from initial problem-statement generation to design, implementation, and evaluation of solutions. In health informatics research, CBPR applies principles borrowed from health equity and disability rights activism of “Nothing about us without us” and “Center the margins” into studies so that the final health technology product does not widen, maintain, or create novel disparities but instead meets the needs of those most affected by social drivers of health and systemic injustice [44,45].

Throughout this viewpoint, we have pointed to challenges in centering LMIC researchers as experts, power sharing, and building strong community relationships, which can all be addressed by combining CBPR and GHI. For example, incorporating the CBPR conceptual model into GHI research can help center traditional and Indigenous knowledge systems, balance action and research, and prompt colearning and coleadership [46,47]. However, by nature, CBPR has no one-size-fits-all approach and will need to be thoughtfully incorporated into GHI. Specifically, capacity building to ensure that researchers and organizations in LMICs can proactively safeguard themselves against exploitation and co-optive CBPR practices, which can foster beneficial long-term relationships and global partnerships.

One caution against leveraging HI lessons from high-income settings and applying them to LMIC contexts is that a constant search for novel and high-performing yet brittle interventions may not be suitable for lower-resource settings. IT solutions are important in all health environments, but local design input, transparency from large IT companies involved in these projects, and local control are crucial to success in diverse environments and cultures. Moving forward, GHI researchers should look to interventions that emphasize commons-based ownership (eg, of GHI interventions), voluntary simplicity, and learning from the traditional wisdom developed and preserved by many cultures globally [23,27]. Post-growth solutions might necessitate a departure from techno-solutionism, an understanding that technology can facilitate solutions, but truly addressing health inequity will require focusing on structural inequities and local innovation in health care delivery [48].

Finally, given longstanding GHI “pilotitis” issues, future research endeavors should focus on leveraging transient grant funding to develop pilots and then help local organizations sustain long-term research. This may look like assistance and support to receive grants supporting postimplementation monitoring, capacity building to ensure that local community members are well-positioned to continue deriving value from knowledge generated by GHI research (eg, entrepreneurship), as well as ensuring principles, such as commons-based ownership and voluntary simplicity. In addition, advocating for policy change is another important avenue to orient grant organizations toward the importance of long-term monitoring and support of successful GHI interventions.

## Conclusions

This viewpoint identified current gaps in the literature on academic GHI implementation and underscored the need for international academic collaborations. A path forward to develop mutually beneficial partnerships and execute projects across the project life cycle phases was outlined, and to circumvent the concept of “pilotitis” was described. The authors propose a call to action to reduce facilitation barriers, strengthen implementation, and GHI knowledge dissemination and translation. This includes developing more appropriate evaluation metrics for GHI interventions outside of publications that encapsulate collaborations’ impact. For example, an open register of digital health systems, such as Digital Square [49], with open data on sites, patients, technology end-users, disease coverage, longevity, etc, is an alternative or complementary metric of the scope and success of academic GHI partnerships compared to publications.

Academic institutions working on GHI interventions could collaborate to establish a global community of practice in which health informatics recommended guidelines and standards could be successfully developed, implemented, adopted, and adapted to suit individual resources (ie, human and financial). This will require more funding and support that accounts for realistic timelines given indirect costs and unique challenges to GHI projects that differentiate them from other health informatics projects in high-income countries. It is critical to reduce barriers to care and implementation through transparent communication, knowledge transfer of lessons learned, and proven techniques to promote sustainable, mutually beneficial GHI academic collaborations. Thus, partnerships between academic institutions and industries spanning diverse countries and geographies are urgently required to address global challenges that transcend national borders without further propagating entrenched inequities [15].

## Abbreviations

CBPR: community-based participatory research
CHOP: Children’s Hospital of Philadelphia
GHI: global health informatics
HIS: health information system
LMIC: low- and middle-income country
mHealth: mobile health
UB: University of Botswana
